# Polygenic risk score and phenome-wide association study of the Epstein-Barr virus antibody response

**DOI:** 10.3389/fgene.2026.1786510

**Published:** 2026-03-16

**Authors:** Bahram Namjou, Michael Lape, Matthew T. Weirauch, Kenneth M. Kaufman, Leah C. Kottyan

**Affiliations:** 1 Center for Autoimmune Genomics and Etiology, Cincinnati Children’s Hospital Medical Center, Cincinnati, OH, United States; 2 Department of Pediatrics, University of Cincinnati College of Medicine, Cincinnati, OH, United States; 3 Division of Human Genetics, Cincinnati Children’s Hospital Medical Center, Cincinnati, OH, United States; 4 Department of Biomedical Informatics, University of Cincinnati College of Medicine, Cincinnati, OH, United States; 5 Division of Allergy & Immunology, Cincinnati Children’s Hospital Medical Center, Cincinnati, OH, United States; 6 Division of Biomedical Informatics, Cincinnati Children’s Hospital Medical Center, Cincinnati, OH, United States; 7 US Department of Veterans Affairs Medical Center, Cincinnati, OH, United States

**Keywords:** EBNA1, EBV, PheWAS, polygenic risk score, serology

## Abstract

**Introduction:**

The Epstein-Barr virus (EBV) infection is nearly ubiquitous and has established links to malignancy and autoimmune disease. Here we evaluate the genetic factors influencing the humoral immune response to EBV and establish a polygenic risk score (PRS) for anti-EBNA1 responses.

**Methods:**

We conducted a multi-biobank genetic study for the serologic humoral IgG antibody response to EBV-EBNA1, including data from the UK Biobank (UKB, N = 9695 individuals) and the Milieu Intérieur (MI) cohort from Institute Pasteur (IP) (N = 1000), as well as GWAS summary statistics for individuals of African ancestry (N = 4365). We divided the cohort into discovery and validation, performed GWAS analyses, and developed a PRS using a Bayesian multi-ancestry approach application (PRS-CSx). After successfully validating PRS predictive performance, we then applied PheWAS analyses to all UKB datasets.

**Results:**

Consistent with a previous report, our GWAS analyses identified an association at chromosome 6 within the MHC region as the most significant SNP (rs6927022; p = 8.21 × 10^−113^). Meta-analyses of all cohorts identified several regions outside of the MHC region with the strongest effects at 10p12 within the armadillo repeat containing three gene (*ARMC3*) (rs10763372; p = 9.93 × 10^−08^) and at 10q22 upstream of Neuregulin-3 (*NRG3*) (rs61851627; p = 8.62 × 10^−9^). A multi-ancestry PRS model was then trained and developed, obtaining successful predictive performance in validation cohorts (AUC ranges from 65% to 72%). Finally, PheWAS analyses using the developed PRS confirmed a significant positive association with multiple sclerosis (p = 1.14 × 10^−10^), and also identified novel negative associations with other spectra of autoimmune disease, including Celiac disease (2.03 × 10^−127^).

**Discussion:**

We developed and validated a multi-ancestry PRS for EBNA1 IgG response that enables genetic profiling of EBV antibody responsiveness in genotyped cohorts lacking serologic measurements. PheWAS results support shared and divergent genetic relationships between EBV antibody response and autoimmune disease, motivating mechanistic and longitudinal follow-up studies. Further research is needed to evaluate the future implementation of this PRS in the clinic to improve long-term health outcomes.

## 1 Introduction

Epstein-Barr virus (EBV) is a ubiquitous human gammaherpesvirus with established roles in malignancy and increasing evidence for causal contributions to select autoimmune diseases. EBV is transmitted primarily through salivary contact and establishes lifelong persistence after primary infection by infecting B cells and maintaining latency within the memory B-cell compartment (Thompson and Kurzrock, 2004). In the United States, EBV seroprevalence rises from approximately 50% in children to nearly 90% in adolescents (Lunn et al., 2017). During latency, the EBV nuclear antigen 1 (EBNA1) is required to maintain the viral episome in proliferating infected B cells. Latency-associated EBV genes, including EBNA1 and latent membrane protein 2A (LMP2A), can support B-cell activation and differentiation programs that, in susceptible contexts, contribute to EBV-associated cancers such as nasopharyngeal carcinoma and Hodgkin lymphoma (Kang and Kieff, 2015).

EBV is also strongly linked to autoimmunity. Multiple studies have demonstrated an association between elevated EBNA1-specific IgG titers and the risk of Multiple Sclerosis (MS) and systemic lupus erythematosus (SLE) (Levin et al., 2005; Laurynenka et al., 2022). Proposed mechanisms include molecular mimicry and cross-reactivity between anti-EBNA antibodies and disease-relevant autoantigens, with potential downstream effects on disease initiation or severity (Laurynenka et al., 2022; Lanz et al., 2022; Tengvall et al., 2019; Thomas et al., 2023). Serologic measures, including antibodies against EBNA1 and viral capsid antigen (VCA), are widely used to characterize host humoral responses to latent infection and viral reactivation, respectively. Acute EBV infection is typically identified by the presence of VCA IgG and VCA IgM in the absence of EBNA1 IgG, while past infection is marked by VCA IgG and EBNA1 IgG without VCA IgM (De Paschale and Clerici, 2012). The highly immunogenic C-terminal domain of EBNA1 (residues 380–641) serves as a key target for host antibodies, making it a critical marker in ELISA assays for identifying past infections, nasopharyngeal carcinoma, and multiple sclerosis (Milman et al., 1985; Sattarnezhad et al., 2025).

Inter-individual variation in EBV antibody responses is partly genetically determined, with prior work estimating substantial heritability for IgG responses that ranges between 32% and 48% in human population (Rubicz et al., 2011; Besson et al., 2009). Genome-wide association studies (GWAS) have consistently implicated the human leukocyte antigen (HLA) class II region as a major contributor to variability in IgG responses, including EBNA1 antibody levels (Rubicz et al., 2013; Hammer et al., 2015). Together, these observations motivate the development of genetic predictors of EBV humoral immunity that can be applied on a scale.

Here, we develop a polygenic risk score (PRS) for EBNA1 IgG responses that estimate an individual’s genetic liability to higher or lower antibody levels. We leverage GWAS data across European and African ancestry populations and apply Bayesian multi-ancestry polygenic modeling to improve portability. We then validate PRS performance in independent cohorts and use phenome-wide association analyses to evaluate clinical and immunologic correlates of genetically predicted EBNA1 response.

## 2 Methods

### 2.1 Study population

We selected participants from the United Kingdom Biobank (UKB), a large long-term biobank cohort that supports the investigation of the respective contributions of genetic predisposition and environmental exposure to the development of diseases (Sudlow et al., 2015). The UK Biobank post-imputed genomic data was obtained through application ID: 47377. This study also received approval to access the Milieu Intérieur (MI) cohort from institute Pasteur (IP) that consists of imputed genotyping data of 1000 healthy European individuals with comprehensive viral serologic information (Scepanovic et al., 2018) (https://www.milieuinterieur.fr/en/). The GWAS summary statistics of the EBV (EBNA1) antibody response in African ancestry (African Cohort, AC) were also obtained from a recent large study and used for multi-ancestry PRS development (Sallah et al., 2020). Details of the study population after quality control are shown in Table 1.

**TABLE 1:** Study population.

Cohorts	Demographic	Discovery	Validation
UKB	Total	6662	1965
	Sex (female/male)	3700/2962	1112/853
	Mean age (SD)	67.9 (8.05)	67.3 (8.30)
	Ancestry	European	Mixed
Milieu Intérieur (MI) cohort from institute Pasteur (IP)	Total	​	954
	Sex (female/male)	​	476/478
	Mean age (SD)	​	44.71 (14.38)
	Ancestry	European	European
African-GWAS summary statistics	Total	4365	​
	Ancestry	African descent (Uganda)	​

Abbreviations: UKB, UK biobank; SD, standard deviation.

### 2.2 Serology

The IgG antibody response to EBV (EBNA1) has been previously measured in 9695 individuals from the UKB population; this dataset was used for GWAS assessment and PRS development (Mentzer et al., 2022). This method provides the median fluorescence intensity (MFI), a standardized quantification of the amount of antibody in the sample obtained by measuring the fluorescence emitted by the analyte-capture agent complex. Validation was performed using separate serum samples and a reference gold standard (Mentzer et al., 2022). In this multiplex methodology, the C-terminal domain (specifically amino acids 325–641), was used to detect high-affinity IgG antibodies against EBNA1, which are highly immunogenic (Brenner et al., 2018). After applying quality control, we divided this cohort into two independent population in which 80% of unrelated individuals of white British ancestry were used for GWAS discovery and PRS development (N = 6662). Furthermore, the GWAS summary statistics of the EBV (EBNA1) antibody response in African ancestry consist of 4365 individuals of African descent, used for multi-ancestry PRS development (Sallah et al., 2020). For validation and predictive performance of the PRS model, we used the remaining 20% individuals from the UKB cohort (n = 1965), as well as a separate cohort of 1000 healthy Europeans individuals from the Milieu Intérieur (MI) cohort from the Institute Pasteur (IP) (https://www.milieuinterieur.fr/en/).

### 2.3 GWAS related analyses

A genome wide association study (GWAS) was performed on UKB subjects with available EBNA1 antibody titer information. The standardized antibody titer was used as a quantitative trait in regression analyses. First, individual genotypic data underwent a series of quality controls in which all analyses were limited to SNPs with call rates >99%, and MAF >1% and HWE p > 0.00001. All participants with sex inconsistencies as well as duplicated or twin individuals, and first-degree relatives were removed using PLINK’s implementation of KING robust kinship coefficients (Chang et al., 2015). After quality control, there were 8,627 individuals used for analyses in the UKB dataset and 954 individuals in the MI cohort (Table 1). Next, quantitative linear regression analyses were performed using an additive genetic model adjusting for 10 Principal Components (PCs), sex; and age using second generation PLINK software (Chang et al., 2015). Finally, a meta-analysis of three post-qc GWAS summary statistics (UKB, MI and African ancestry GWAS) was performed using the inverse variance weighted method implemented in PLINK (Chang et al., 2015). For visualization of specific effects, Zoom plots were generated using LocusZoom (http://csg.sph.umich.edu/locuszoom). To facilitate functional annotation of GWAS results and gene prioritization, significant variants from association analyses were mapped to genes using the FUMA platform tool (https://fuma.ctglab.nl/) with default parameters (Watanabe et al., 2017).

### 2.4 Polygenic risk score development and performance evaluation

A polygenic risk score was developed and validated using established computational tools (PLINK and PRS-CSx) that jointly model the GWAS summary statistics from different ancestries and couple genetic effects across populations using a shared continuous shrinkage prior (Ruan et al., 2022). In this approach, the program infers effect sizes of genetic variants using discovery GWAS summary statistics and accounts for linkage disequilibrium using an external reference panel (i.e., the Phase 3 release of the 1000 Genomes data). At the next step, the population-specific posterior effect-size estimates are combined using an inverse-variance-weighted meta-analysis within the Gibbs sampler. The final PRS-CSx output included 723164 HapMap3 variants and their posterior weights. For PRS performance estimation and validation, we selected the top 10% of the EBNA1 standardized distribution as a threshold for binary phenotypes of cases and the bottom 10% as controls. PRS prediction accuracy and performance was then assessed using the Area Under the Receiver Operating Curve (AUROC), odds ratio per 1 SD (Standard Deviation) (OR), and by the amount of variance explained (R2) after accounting for covariates that includes 10 principal components, age, and sex. In addition, the developed PRS was also used to assess the EBV seropositivity prediction accuracy according to criteria from the manufacturers (Mentzer et al., 2022; Scepanovic et al., 2018). The seropositivity rate was 94% in the MI cohort and 95% in the UKB cohort, consistent with the general population.

### 2.5 PheWAS analyses

A phenome-wide association study (PheWAS) was also performed in order to evaluate pleotropic effects of the developed PRS with any other phenotypic trait. We used the PheWAS package in R version 3.5.1 (Carroll et al., 2014). Briefly, in the PheWAS process, first the ICD codes are collapsed into phecodes according to the PheWAS map. Then, cases and controls are determined according to the criteria under study. In these analyses, a case was defined as having at least two occurrences of the PheWAS code on different days. Controls had no instances. Additionally, we used a threshold of at least 20 cases for the code to be used in the model. Next, for each PheWAS code, a logistic regression model was created and adjusted for age, sex, and PCs similar to the GWAS study. A false discovery rate (FDR) of 0.05 using the Benjamini–Hochberg procedure implemented in PheWAS was then used to correct the threshold for multiple hypotheses testing.

## 3 Results

**FIGURE 1:**
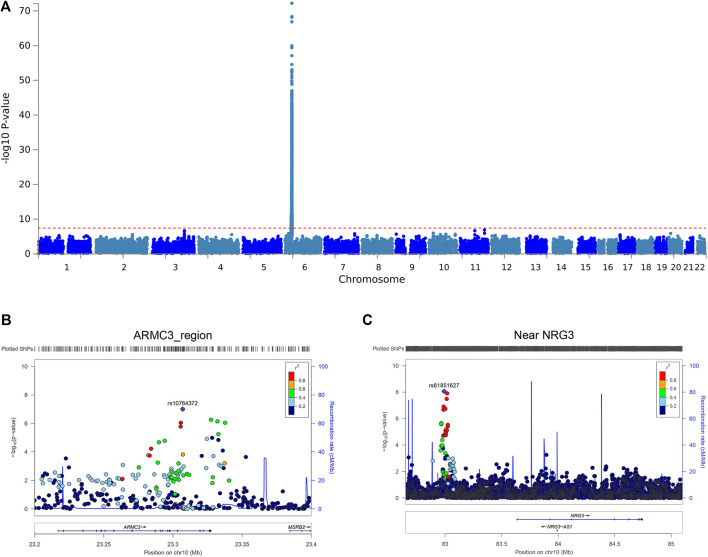
**(A)** Manhattan plot of genome-wide markers for EBNA1 serum IgG levels in the UKB cohort. The major effect within the HLA region at Chromosome 6 is shown with no genetic inflation (inflation 1.0). **(B,C)** LocusZoom plots of association signals, outside of the HLA region, at Chromosome 10 (*ARMC3* and *NRG3*), after meta-analyses of all three cohorts.

In genome-wide association analyses of EBNA1 IgG levels, the strongest association signal localized to the HLA/MHC region (Figure 1A). In the European-ancestry analysis, rs6927022 showed the strongest association (p = 8.02 × 10^−73^; β = 0.32; SE = 0.01). This same variant was also strongly associated in the African-ancestry GWAS (p = 1.36 × 10^−21^), and the cross-cohort meta-analysis yielded a combined p-value of 8.21 × 10^−113^, consistent with a shared association signal across ancestries. Several imputed HLA alleles in UK Biobank were in linkage disequilibrium with rs6927022 and also showed strong association, including HLA-DQA1*0102* (p = 2.45 × 10^−32^) and *HLA-DRB1*1501 (p = 4.14 × 10^−27^) (Supplementary Table S1).

Outside the MHC, association signals were predominantly suggestive. However, in the meta-analysis across post-QC GWAS summary statistics (see Methods), we observed two loci on chromosome 10 that reached or approached genome-wide significance (p < 5 × 10^−8^) (Figures 1B,C). The strongest non-MHC signal was upstream of *NRG3* at 10q22 (rs61851627; p = 8.62 × 10^−9^; Figure 1B). A second signal was observed at 10p12 within *ARMC3* (intronic rs10763372; p = 9.93 × 10^−8^; Figure 1C). For both loci, there was no evidence of heterogeneity across cohorts (Cochran’s Q p ≥ 0.05; Supplementary Table S1). Across non-MHC regions, 72 variants met a suggestive threshold (p ≤ 1 × 10^−5^). Gene-based and functional mapping prioritized 14 genes outside the MHC, including *ARMC3* and *NRG3*. Pathway enrichment of prioritized non-MHC genes nominated several biological processes, with the most significant signal in a purinergic nucleotide receptor signaling pathway gene set (MsigDB c5; p = 7.44 × 10^−8^) (Supplementary Table S1).

We next developed a multi-ancestry polygenic risk score using PRS-CSx with European- and African-ancestry discovery GWAS summary statistics. The final model included 723,164 HapMap3 variants with posterior weights (Supplementary Table S1). In validation analyses, we evaluated prediction of EBNA1 IgG extremes by contrasting the top 10th percentile versus the bottom 10th percentile of the standardized EBNA1 IgG distribution.

**FIGURE 2:**
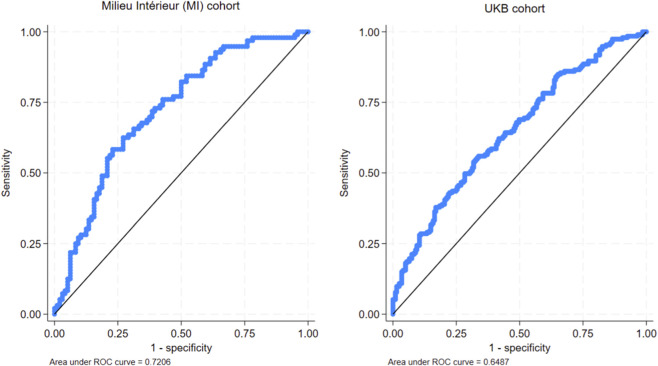
Area Under the Curve (AUC) as a function of PRS predictive performance in the Milieu Intérieur (MI) cohort (AUC = 0.72) and UKB cohort (AUC = 0.65). (see Supplementary Table S1 for more details).

In the UK Biobank validation subset (N = 1,965), the PRS achieved an AUC of 0.65 (95% CI 0.60–0.70) in a logistic model adjusted for age, sex, and 10 principal components. This corresponded to ∼6% variance explained (R^2^ = 0.06) and an odds ratio of 1.56 (95% CI 1.24–1.95) for case status under the extreme-quantile definition. In the Milieu Intérieur cohort (N = 954), performance was higher (AUC = 0.72; R^2^ = 0.11), with an odds ratio per 1 SD increase of 1.50 (95% CI 1.06–2.09) (Figure 2; Supplementary Table S1). We also evaluated whether the PRS predicted EBV seropositivity status. The PRS predicted seropositivity in both cohorts (AUC = 0.67 [95% CI 0.61–0.72] in UK Biobank; AUC = 0.64 [95% CI 0.57–0.71] in Milieu Intérieur), noting that seronegative individuals comprised <5% of each cohort (See Methods).

To evaluate phenotypic correlates of genetically predicted EBNA1 IgG response, we performed a PheWAS in UK Biobank using the standardized PRS, adjusted for age, sex, and 10 principal components, with multiple testing control at FDR<0.05. The ten most significant associations are summarized in Table 2 (see Supplementary Table S1 for full results). The PRS showed a significant positive association with multiple sclerosis (p = 1.14 × 10^−10^), consistent with prior epidemiologic links between EBV antibody responses and MS. In contrast, we observed strong inverse associations with several autoimmune phenotypes, including celiac disease (p = 2.03 × 10^−127^) and type 1 diabetes (Table 2; Supplementary Table S1).

**TABLE 2:** Top10 significant PheWAS effects in the UKB dataset.

Description	Cases	Controls	p	OR	Lower	Upper
Celiac disease	2890	282654	2.03E-127	0.64	0.62	0.67
Disorders of iron metabolism	1240	396214	5.36E-52	1.52	1.44	1.61
Type 1 diabetes	4349	363177	1.58E-25	0.85	0.83	0.88
Type 1 diabetes with ophthalmic manifestations	806	363177	4.78E-16	0.75	0.70	0.80
Disorders of mineral metabolism	7054	396214	4.93E-15	1.10	1.07	1.13
Type 1 diabetes with ketoacidosis	424	363177	1.20E-12	0.71	0.64	0.78
Hypothyroidism NOS	25668	372003	5.81E-11	0.96	0.95	0.97
Hypothyroidism	26987	372003	1.10E-10	0.96	0.95	0.97
Multiple sclerosis	1860	375727	1.14E-10	1.16	1.11	1.21
Diabetes mellitus	38368	363177	7.47E-09	0.97	0.96	0.98

To contextualize these genetic associations with measured serology, we compared standardized EBNA1 IgG levels across autoimmune phenotypes in UK Biobank. Consistent with the PheWAS, individuals with MS had higher mean EBNA1 IgG levels (mean = 0.53), whereas individuals with celiac disease had lower mean levels (mean = −0.31, P < 0.0001). Additional autoimmune phenotypes showed directionally consistent patterns, including higher mean EBNA1 IgG levels in ankylosing spondylitis, inflammatory bowel disease, and rheumatoid arthritis, and lower levels in type 1 diabetes (Supplementary Table S1).

## 4 Discussion

We developed and validated a multi-ancestry polygenic risk score (PRS) for EBNA1 IgG antibody response by leveraging PRS-CSx to integrate GWAS summary statistics across diverse populations. Our approach involved four steps: (a) constructing the PRS using serological data; (b) validating the score in an independent cohort; (c) projecting weights onto the full UK Biobank (UKB) population; and (d) conducting phenome-wide association studies (PheWAS) with stringent multiple testing correction. This study provides a robust, scalable genetic proxy for EBNA1 response, enabling analysis in large cohorts lacking serological data.

Consistent with prior work on host genetic control of EBV humoral responses (Rubicz et al., 2011; Besson et al., 2009; Mentzer et al., 2022; Sallah et al., 2020), we observed that the dominant GWAS signal localizes to the HLA/MHC region. While this study primarily aimed to develop EBNA1 PRS across all resources rather than focus on GWAS discovery, our meta-analysis unexpectedly revealed novel, non-HLA, effects that have not been previously described. These include two signals at 10p12 (near *ARMC3*) and 10q22 (upstream of *NRG3*) that reached or approached genome-wide significance (Figures 1B,C). These associations are best interpreted as hypotheses that prioritize biological follow-up rather than definitive mechanistic assignments. Prior links between the *ARMC3* region and IgG glycosylation traits motivate targeted evaluation of whether EBNA1 antibody features (including glycosylation state and effector potential) mediate the observed genetic associations (Lauc et al., 2013). Similarly, the *NRG3*-proximal signal may reflect regulatory effects that are not yet well characterized in the context of antibody response, and functional fine-mapping will be required to identify the causal variants, tissue contexts, and effector genes.

The PRS demonstrated reproducible discrimination of EBNA1 IgG extremes in two independent validation cohorts, with AUCs in the 0.65–0.72 range using an extreme-quantile design. While performance is not sufficient for stand-alone clinical prediction, it is well-suited for research applications that require stratification of large cohorts by genetically predicted EBNA1 response, enrichment of samples for mechanistic studies, or inclusion of EBNA1 response as a covariate or effect modifier in gene–environment analyses. The same PRS also predicted EBV seropositivity, although seronegative individuals represented a small fraction of both validation cohorts, which limits inference about performance for EBV infection status.

PheWAS analyses of the EBNA1 PRS recapitulated the well-described positive relationship between EBV antibody response and multiple sclerosis (Bjornevik et al., 2022) supporting the biological relevance of the genetic proxy. Notably, we also observed inverse associations with several autoimmune phenotypes, particularly celiac disease and type 1 diabetes, and these directions were concordant with comparisons using measured EBNA1 titers in UK Biobank. These findings highlight that genetically predicted EBNA1 antibody response is not uniformly aligned with autoimmune risk and instead may reflect a mixture of shared HLA-driven effects, disease-specific immune architectures, and differences in how EBV-related immune responses interact with distinct pathways of autoimmunity.

Importantly, PheWAS associations do not establish causality; additional studies will be needed to disentangle correlation from mechanistic contribution and to evaluate whether these relationships are mediated through EBV-specific immunity, broader immune responsiveness, or correlated genetic factors. Additionally, phenotypes derived from biobank and EHR resources can be subject to heterogeneity and misclassification, and replication of the strongest PheWAS findings in independent biobanks will be important.

In summary, this work establishes a validated multi-ancestry PRS for EBNA1 IgG response and demonstrates how PRS-based PheWAS can be used to interrogate broad clinical correlates of host humoral responses to EBV. By enabling indirect profiling of EBV antibody response in cohorts without serology, this PRS provides a practical tool for studies of gene by environment interaction, immune endophenotypes, and the links between viral response and autoimmunity.

## Data Availability

The original contributions presented in the study are included in the article/Supplementary Material, further inquiries can be directed to the corresponding author.
